# Comparative analysis of human Wharton’s jelly mesenchymal stem cells derived from different parts of the same umbilical cord

**DOI:** 10.1007/s00441-017-2699-4

**Published:** 2017-12-04

**Authors:** Dinesh Bharti, Sharath Belame Shivakumar, Ji-Kwon Park, Imran Ullah, Raghavendra Baregundi Subbarao, Ji-Sung Park, Sung-Lim Lee, Bong-Wook Park, Gyu-Jin Rho

**Affiliations:** 1 Department of Theriogenology and Biotechnology, College of Veterinary Medicine, Gyeongsang National University, Jinju, Republic of Korea; 2 Department of Obstetrics and Gynecology, School of Medicine, Gyeongsang National University, Jinju, Republic of Korea; 3 Department of Oral and Maxillofacial Surgery, School of Medicine, Gyeongsang National University, Jinju, Republic of Korea; 4 Research Institute of Life Sciences, Gyeongsang National University, Jinju, Republic of Korea

**Keywords:** Transdifferentiation, Hepatocytes, Mesenchymal stem cells, Wharton’s jelly, Umbilical cord

## Abstract

Easy isolation, lack of ethical issues, high proliferation, multi-lineage differentiation potential and immunomodulatory properties of umbilical cord (UC)-derived mesenchymal stem cells (MSCs) make them a valuable tool in stem cell research. Recently, Wharton’s jelly (WJ) was proven as the best MSC source among various compartments of UC. However, it is still unclear whether or not Wharton’s jelly-derived MSCs (WJMSCs) from different parts of the whole cord exhibit the same characteristics. There may be varied MSCs present in different parts of WJ throughout the length of the UC. For this purpose, using an explant attachment method, WJMSCs were isolated from three different parts of the UC, mainly present towards the placenta (mother part), the center of the whole cord (central part) and the part attached to the fetus (baby part). WJMSCs from all three parts were maintained in normal growth conditions (10% ADMEM) and analyzed for mesenchymal markers, pluripotent genes, proliferation rate and tri-lineage differentiation potential. All WJMSCs were highly proliferative, positively expressed CD90, CD105, CD73 and vimentin, while not expressing CD34, CD45, CD14, CD19 or HLA-DR, differentiated into adipocytes, osteocytes and chondrocytes and expressed pluripotency markers OCT-4, SOX-2 and NANOG at gene and protein levels. Furthermore, MSCs derived from all the parts were shown to have potency towards hepatocyte-like cell differentiation. Human bone marrow-derived MSCs were used as a positive control. Finally, we conclude that WJMSCs derived from all the parts are valuable sources and can be efficiently used in various fields of regenerative medicine.

**Electronic supplementary material:**

The online version of this article (10.1007/s00441-017-2699-4) contains supplementary material, which is available to authorized users.

## Introduction

The present era of stem cell biology has become highly advanced in the field of cell therapy, due to the evaluation of various stem cell sources producing fascinating results. After isolation of bone marrow mesenchymal stem cells (BM-MSCs) by Friedenstein et al. (1970), various attempts have been tried to isolate MSCs from different sources, such as adipose (Choi et al. 2015), dental (Ullah et al. 2016, Park et al. 2014), placenta (Li et al. 2005), amniotic fluid (Ghionzoli et al. 2013), umbilical cord (UC) (Xu et al. 2014) and Wharton’s jelly (WJ) (Fong et al. 2010).

Easy isolation, lack of ethical issues and the presence of both embryonic and adult MSC characteristics have made WJ a valuable source and its multi-differentiation potential, immunomodulatory features and long-term survivability without undergoing senescence provide researchers with a unique choice for their use in therapeutic applications. This alternative source of MSCs was first described by Thomas Wharton in 1656 and came into existence when McElreavey reported the culture of WJ-derived cells that is the primitive connective tissue of human UC (McElreavey et al. 1991). The UC, which serves as a link between mother and the developing fetus, contains a variety of mesenchymal stem cell sources, namely umbilical cord blood (UCB), WJ, perivascular regions surrounding the vessels, arteries, veins, the media and the adventitia compartment of the walls of UCB vessels (Kim et al. 2013; Bongso and Fong 2013). Therefore, researchers have tried to compare different compartments of the UC to find the best MSC source among them. Comparative analysis of arterial (UCA), venous (UCV) and WJ explant-derived MSCs for their sustained proliferation and multi-differentiation potential have shown interesting results (Ishige et al. 2009).

More recently, another study reported immunologic and hematopoietic profiles of MSCs derived from different sections of human UC. Human UC (UC), amniotic membrane (AM), WJ and umbilical vessels derived MSCs showed similar biological characteristics including CD marker expression and multi-differentiation potential (Xu et al. 2014). These studies confirm the isolation of various MSC sources from different regions of UC as well as (Wang et al. 2004) from the WJ. Wharton’s Jelly, a gelatinous substance, is responsible for preventing umbilical vessels from compression, torsion and bending (Kim et al. 2013).

As per the previous theories, MSCs are supposed to be trapped in the gelatinous WJ during early human development (Wang et al. 2008; Taghizadeh et al. 2011). Therefore, MSCs must reside in the WJ throughout the cord length irrespective of location, i.e., towards the mother’s side or apart from it. Recently, Subramanian and colleagues represented WJ as a best source of MSCs among other regions of the UC such as the amnion epithelial membrane (AM), the sub-amnion (SA) and the perivascular region (PV) (Subramanian et al. 2015). Although various studies have reported mesenchymal characterization from different regions of human UC, so far nobody has characterized MSCs from different parts of the WJ, namely the mother, baby and central attachment regions. Moreover, differently abled MSCs could be present in different parts. Therefore, the present study focuses on the characterization of WJMSCs derived from different parts. These WJMSCs derived from mother, baby and central parts of the UC have been termed MBC-WJMSCs in the present study. This is the first study of its kind showing the hepatocyte-like cell (HLC) differentiation potential of MSCs derived from various parts of Wharton’s jelly. Multi-differentiation potential of WJMSCs towards neurons (Liang et al. 2013), pancreatic cells (Wu et al. 2009), male germ-like cells (Huang et al. 2010), retinal progenitor cells (Hu et al. 2013) and hepatocytes (Prasajak and Leeanansaksiri 2013; Shivakumar et al. 2016) have been reported by many studies but their endodermal differentiation from different parts has not been fully elucidated until now.

Therefore, the present study aims to find the answers to a few questions concerning whether all parts containing WJ throughout the length of the UC have the same stemness features or which part constitutes the best among them on the basis of their basic characterization along with their hepatocyte (endodermal) differentiation potential.

## Materials and methods

### Chemicals and media

All chemicals were purchased from Sigma (St. Louis, MO, USA) and media from Gibco (Invitrogen, Burlington, ON, Canada), unless otherwise specified.

### Isolation and culture of human Wharton’s jelly-derived MSCs

**Fig. 1:**
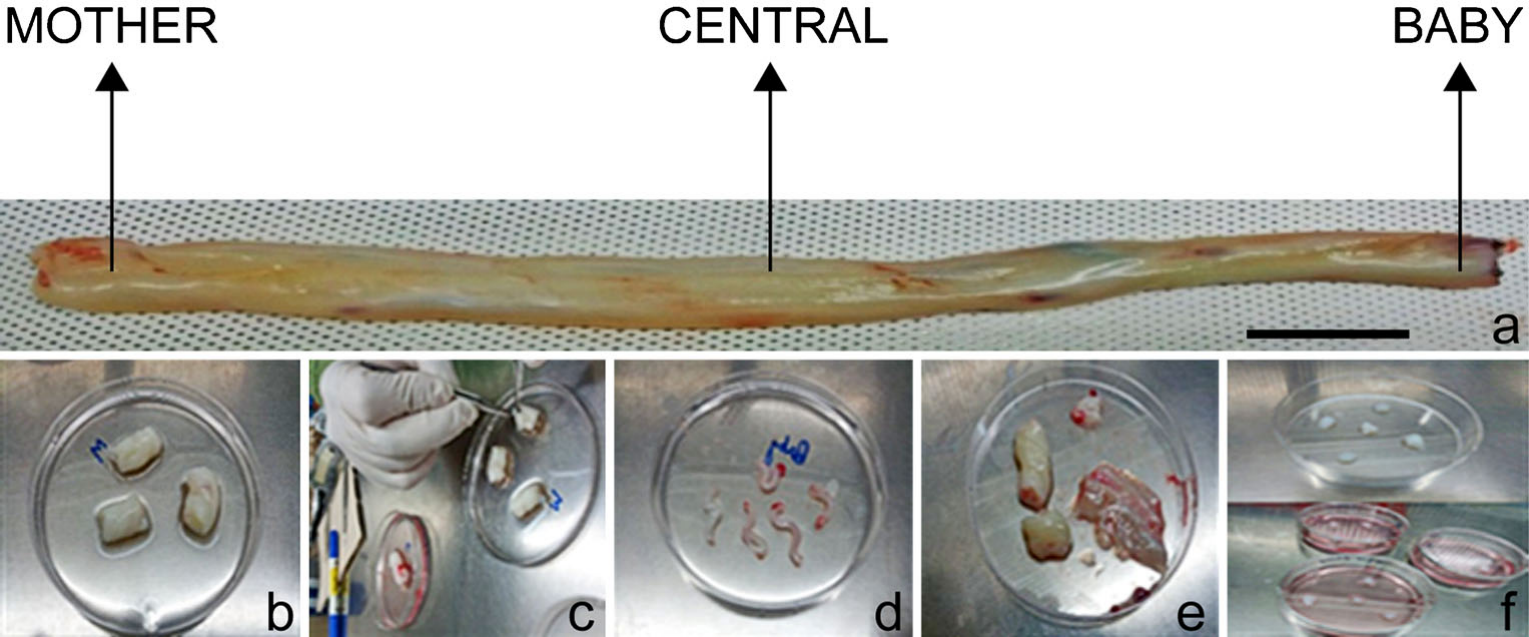
Isolation of MBC-WJMSCs from an umbilical cord. **a** The mother, central and baby regions of a full-length umbilical cord. **b** Cutting of cord pieces. **c** Surgical removal of vessels by exposing Wharton’s jelly (WJ). **d** Removed vessels. **e** Exposed WJ. **f** Explant pieces attached to ADMEM-coated and pre-incubated plates

Human UCs in 1-to 2-cm pieces from three different regions present towards the placenta (the mother part), the center of the full cord length (the central part) and towards the baby region (the baby part) were aseptically collected from the delivery room in Gyeongsang National University Hospital (GNUH) after obtaining informed consent from the donors (*n* = 5) and approval by the Ethic Committee for Clinical Research at GNUH (GNHUIRB-2009-004). MSCs were obtained from samples as per previously described protocols with minor modifications (Fong et al. 2010; Majore et al. 2011). Briefly, after washing with Dulbecco’s phosphate-buffered saline (DPBS) 2–3 times, the UC pieces were carefully dissected to remove vessels and then cut into 1-mm pieces followed by their attachment with Advanced Dulbecco’s modified Eagle medium (ADMEM) supplemented with 10% fetal bovine serum (FBS) (10% ADMEM) to coated and pre-incubated 6-cm plates. The explant-seeded plates were kept under incubation for 1–2 h in the inverted position to ensure proper attachment of the explants. Afterwards, 1–2 ml of 10% ADMEM medium was gently added to the seeded plate followed by incubation at 37 °C in a humidified 5% CO_2_ incubator (Fig. 1a–f). An outgrowth of adherent cells from the tissue explants was observed after 1 week of attachment. After 10–12 days, the tissue explants were removed from the petri dishes leaving behind the attached cells. All the petri dishes were kept in controlled incubation conditions at 37 °C in 5% CO_2_ in air atmosphere. Media were changed twice a week. Isolated cells showed the formation of colonies with fibroblast morphology within 2 weeks of isolation. At 80% confluence, cell harvesting was carried out using 0.25% (w/v) trypsin-EDTA (Gibco), followed by centrifugation at 300*g* for 5 min. Harvested cells were grown to passage 3 for all the experiments. An inverted phase contrast microscope (Nikon DIAPHOT 300, Japan) was used to monitor the phenotype and growth behavior of the primary and passaged cells and photographs were taken for further analysis.

### CD and pluripotent markers expression analysis

At the third passage, pluripotency marker analysis of MBC-WJMSCs was carried out at mRNA and protein level. Briefly, after isolating RNA and cDNA synthesis, PCR amplification was carried out by using Maxime PCR Premix (iNtRON Biotechnology, Seongnam, Korea) and samples were run in Eppendorf Master Cycler (Eppendorf, Hamburg, Germany). The PCR product was confirmed by using gel electrophoresis. WJMSCs from all the three parts were used for cell surface marker expression using flow cytometry (BD FACS Calibur; Becton Dickinson, Franklin Lakes, NJ, USA) in triplicate. Cells at 80~90% confluence were harvested and fixed with 3.7% formaldehyde solution until further processing. After being washed twice with DPBS, cells (1 × 10^5^ cells per marker; Choi et al. 2015) were directly labeled with fluorescein isothiocyanate (FITC)-conjugated CD45 (FITC Mouse Anti-Human CD45; Santa Cruz Biotechnology), CD34 (FITC Mouse Anti-Human CD34; BD Pharmingen, CA, USA), CD90 (FITC Mouse Anti-Human CD90; BD Pharmingen) and unconjugated CD105 (Mouse monoclonal IgG2a; Santa Cruz Biotechnologies), CD73 (Mouse monoclonal; Santa Cruz Biotechnologies), Vimentin (Mouse monoclonal; Sigma), CD14 (Mouse monoclonal; Santa Cruz Biotechnologies), CD19 (Mouse monoclonal; Santa Cruz Biotechnologies) and HLA-DR (Mouse monoclonal; Santa Cruz Biotechnologies) for 30 min. Secondary FITC-conjugated goat anti-mouse IgG (BD Pharmingen) was used for the treatment of unconjugated primary antibodies for 30 min in the dark, whereas Mouse IgG1 (BD Pharmingen) was used as the isotype-matched negative control.

### Cell proliferation and population doubling time

The proliferation capacity of WJMSCs was evaluated by population doubling time (PDT). Briefly, WJMSCs from all three parts at passage 3 were used to assess their proliferation rate. A total of 5 × 10^3^ cells were seeded in triplicate in each well of the 24-well culture plates. Cell number was determined for 13 days at 2-day intervals. The PDT of all experimental groups was calculated by using a formula, PDT = *t* (log2) / (logNt-logN0), where N_O_ and N_*t*_ are the initial and final numbers of MSCs before and after seeding, respectively, whereas *t* represents the culture time.

### In vitro differentiation capacity

At passage 3, ~70% confluent WJMSCs from all three parts were seeded into 6-well plates at a density of 7.5 × 10^4^ cells/well and induced to adipogenic, osteogenic and chondrocyte lineages by culturing under suitable conditions for 21 days following the previously published protocol (Park et al. 2014). Human BMMSCs were also induced to tri-lineage differentiation using same lineage-specific culture conditions and were taken as a positive control. Adipogenic medium consisted of ADMEM supplemented with medium 1 mM dexamethasone, 100 mM indomethacin, 10 mM insulin and 500 mM isobutyl methyl xanthine, while osteogenic medium consisted of ADMEM supplemented with 0.1 mM dexamethasone, 10 mM glycerol-2-phosphate and 50 mM ascorbate-2-phosphate. Cells were induced to chondrogenic lineage by using commercial chondrogenic medium (StemPro1Osteocyte/Chondrocyte Differentiation Basal Medium; StemPro1Chondro-genesis supplement; Gibco). Successful differentiation was evaluated by staining the differentiated cells with Oil red O, von Kossa and alizarin red, Alcian blue and Safranin O in cases of differentiated adipocytes, osteocytes and chondrocytes, respectively. Untreated cells were also stained with these stains. Media were changed at 2-day intervals.

### Endodermal differentiation (hepatocytes)

Hepatogenic differentiation potential of WJMSCs and BMMSCs was performed as previously described (Patil et al. 2014; Shivakumar et al. 2016). Initially, WJMSCs and BMMSCs were grown in 10% FBS-supplemented ADMEM until 70% confluence and further induced with hepatocyte culture media consisting of 2% FBS-enriched ADMEM with 20 ng/ml recombinant human hepatocyte growth factor (HGF; R&D Systems, MN, USA) for 7 days. Furthermore, primarily treated WJMSCs and BMMSCs were cultured with hepatocyte maturation medium comprised of ADMEM supplemented 2% FBS, 10 ng/ml of oncostatin M (R&D Systems), 10 nmol/l dexamethasone and 1% insulin-transferrin-selenium mix for 15 days. WJMSCs and BMMSCs grown in 10% FBS containing ADMEM without any chemical treatment were taken as control. Media were changed twice a week.

#### Immunocytochemistry

For immunocytochemical staining, cells were initially fixed with 3.7% formaldehyde for 1 h and permeabilized with 0.25% Triton X-100 for 10 min at room temperature. After blocking with 1% bovine serum albumin in DPBS for 1 h, the cells were incubated overnight with primary antibodies, such as goat anti-human serum albumin (ALB; 1:200; Santa Cruz Biotechnology) and goat anti-hepatocyte nuclear factor 1-alpha (HNF-1α; 1:200; Santa Cruz Biotechnology) at 4 °C followed by incubation with CruzFluor™ 594 conjugated donkey anti-goat IgG (1:200; Santa Cruz Biotechnology) and CruzFluor™ 488 conjugated donkey anti-goat IgG (1:200; Santa Cruz Biotechnology) secondary antibody for 45 min at 37 °C, respectively. For counterstaining of cells nuclei 1 μg/ml 4′, 6-diamidino-2-phenylindole was used for 5 min and corresponding images were taken using a fluorescent microscope (Leica, Wetzlar, Germany). Untreated cells were also stained with corresponding antibodies to confirm that during immunostaining the detected signal in the differentiated cells was not due to background auto-fluorescence.

#### Periodic acid-Schiff (PAS) staining

Differentiated hepatocytes from all the experimental groups were evaluated for their glycogen storage ability using PAS staining. Briefly, both control and differentiated types of cells were initially fixed in 3.7% formaldehyde for 1 h followed by washing with DPBS three times. Furthermore, cells were treated with an oxidizing agent 1% periodic acid for 5 min at room temperature and rinsed with distilled water for 5–10 min before treating with Schiff’s reagent for 15 min at room temperature. Finally, both types of cells were counter-stained with Mayer’s hematoxylin for 30 s and washed with distilled water to remove the excess stain. Glycogen storage was observed under a light microscope.

#### Urea assay

Both control and differentiated cells were incubated with 1 mM ammonium chloride (NH_4_Cl) containing culture medium for 24 h. Cell supernatants were collected, centrifuged at 300*g* for 5 min and urea levels were measured in 96-well plates at 570 nm as per the manufacturer’s instruction manual (Abcam, Cambridge, MA, USA). Culture medium supplemented with 1 mM NH_4_Cl was used as a control.

#### Low-density lipoprotein (LDL) uptake assay

LDL uptake by hepatocyte differentiated WJMSCs and BMMSCs was evaluated by using Dil AcLDL (low-density lipoprotein from Human Plasma, Acetylated, Dil complex; Thermo Fisher Scientific, MA, USA). Briefly, cell incubation was carried out in serum-free DMEM-LG medium supplemented with 10 μg/ml Dil AcLDL for 4 h at 37 °C. Finally, cells were washed with DPBS and visualized under a fluorescent microscope.

## Western blotting

Protease inhibitor containing RIPA buffer (Thermo Scientific, Rockford, IL, USA) was used to prepare protein lysate from control and differentiated cells from all the experimental groups. Protein concentration was evaluated by using Microplate BCA Protein Assay kit (Pierce Biotechnology, Rockford, IL, USA). A total of 25 μg of each protein sample were separated by 12% sodium dodecyl sulfate-polyacrylamide gel electrophoresis (Mini Protean; BioRad, Hercules, CA, USA) and transferred onto polyvinylidene difluoride membranes (Millipore, USA). Furthermore, membrane incubation was carried out with primary antibodies such as rabbit anti-Sox-2 (1:200; Santa Cruz Biotechnology), goat anti-Nanog (1:200; Santa Cruz Biotechnology), goat anti-Oct-3/4 (1:200; Santa Cruz Biotechnology), goat HNF-1α (1:200; Santa Cruz Biotechnology), rabbit anti-β actin (1:1000; Cell Signaling Technology) and ALB (1:200; Santa Cruz Biotechnology) overnight at 4 °C followed by incubation with horseradish peroxidase-conjugated goat anti-rabbit IgG (1:10,000; Santa Cruz Biotechnology) and donkey anti-goat IgG (1:10,000; Santa Cruz Biotechnology) secondary antibodies at room temperature for 1 h. Finally, enhanced chemiluminescence (Supersignal, West Pico Chemiluminescent substrate; Pierce, IL, USA) was used for the detection of immunoreactivity on exposure to X-ray films. A complete description of the antibodies, their concentrations used along with their manufacturer details is shown in Table S1.

## RNA extraction – cDNA synthesis – RT-qPCR

Total RNA from all the experimental groups (both control and differentiated cells) was isolated using the RNeasy mini kit (Qiagen, Valencia, CA, USA) following the manufacturer’s protocol with the additional elimination of genomic DNA. The concentration and purity of total RNA were determined by measuring the optical density at 260 nm and a 260 nm/280 nm ratio, respectively. With the help of Omniscript RT kit (Qiagen) with oligo-dT primer, complementary cDNA was prepared from a total 2 μg RNA and the reaction was carried out at 37 °C for 60 min. For the analysis of expressed transcription factors, lineage-specific marker genes, real-time PCR was carried out on a Rotor gene Q (Qiagen) using Rotor Gene™ SYBR green PCR kit (Qiagen). Briefly, a 25-μl reaction volume was formulated using a total of 50 ng cDNA mixed with 12.5 μl SYBR Green mix, 5.5 μl RNase-free water and 1 μl each of forward and reverse primers. Following the manufacturer’s instructions, the assay was performed with initial denaturation at 95 °C for 10 min, followed by 40 PCR cycles of 95 °C for 10 s, 60 °C for 6 s and 72 °C for 4 s, followed by a melting curve from 60 to 95 °C at 1 °C/s and then cooling at 40 °C for 30 s. Rotor-Gene Q series software (Qiagen) was used to analyze CT value melting curves of each sample. Normalization of gene expression for all the experimental groups was carried out by using YWHAZ (tyrosine 3-monooxygenase/tryptophan 5-monooxygenase activation protein, zeta polypeptide) as a housekeeping gene. The PCR products were evaluated by 1.5% agarose gel electrophoresis and images were analyzed using zoom browser EX5.7 software (Canon) and the relative level of gene expression was calculated according to the 2^-ΔΔCT^ method. The primers used are listed in Table 1. All experiments were carried out in triplicate.

**Table 1:** List of primers used in the gene expression profiling of WJMSCs by using RT-PCR

Gene	Primer sequence	Product size (bp)	Accession no.
*OCT4*	F: AAGCAGCGACTATGCACAAC R: AGTACAGTGCAGTGAAGTGAGG	140	NM_002701.5
*SOX2*	F: CACCCACAGCAAATGACAGC R: AGTCCCCCAAAAAGAAGTCCAG	120	NM_003106.3
*NANOG*	F: GCAGATGCAAGAACTCTCCAAC R: CTGCGTCACACCATTGCTATTC	133	AB093576.1
*RUNX-2*	F: ATGTGTGTTTGTTTCAGCAG R: TCCCTAAAGTCACTCGGTAT	199	NM_001024630.3
*OSTEONECTIN*	F: GTGCAGAGGAAACCGAAGAG R: AAGTGGCAGGAAGAGTCGAA	202	J03040.1
*BMP2*	F: TAGACCTGTATCGCAGGCAC R: GGTTGTTTTCCCACTCGTTT	149	NM_001200.2
*PPARγ*	F: TTGCTGTCATTATTCTCAGT R: GAGGACTCAGGGTGGTTCAG	124	AB565476.1
*FABP4*	F: TGAGATTTCCTTCATACTGG R: TGGTTGATTTTCCATCCCAT	128	NM_001442.2
*CEBPα*	F: GAAGTCGGTGGACAAGAAC R: CATTGTCACTGGTCAGCTC	140	NM_004364.4
*SOX9*	F: ATGGAGCAGCGAAATCAACG R: CAAAGTCCAAACAGGCAGAGAG	118	BC007951.2
*AGGRECAN*	F: GAATGGGAACCAGCCTATACC R: TCTGTACTTTCCTCTGTTGCTG	98	NM_001135.3
*COLLAGEN-II*	F: GAGACCTGAAACTCTGCCACC R: TGCTCCACCAGTTCTTCTTGG	165	NM_001844.4
*ALB3*	F: CAGGCGACCATGCTTTTCAG R: TTATCGTCAGCCTTGCAGCA	243	NM_000477.5
*HNF4A1*	F: GGAAGTGGCTGAGTCAGGAC R: CGGAAGCCCCTCAACTTGAT	129	NM_178849.2
*YWHAZ*	F: ACGAAGCTGAAGCAGGAGAAG R: TTTGTGGGACAGCATGGATG	111	BC108281.1

## Statistical analysis

All experimental groups were analyzed for their statistical differences by one-way ANOVA using SPSS 21.0. All data are presented as mean ± standard error of the mean and Tukey’s test was performed to present the data from multiple comparisons. All experiments were performed in triplicate. Values of *P* < 0.05 were considered significant.

## Results

### Mesenchymal characteristics from mother, central and baby parts of Wharton’s jelly

**Fig. 2:**
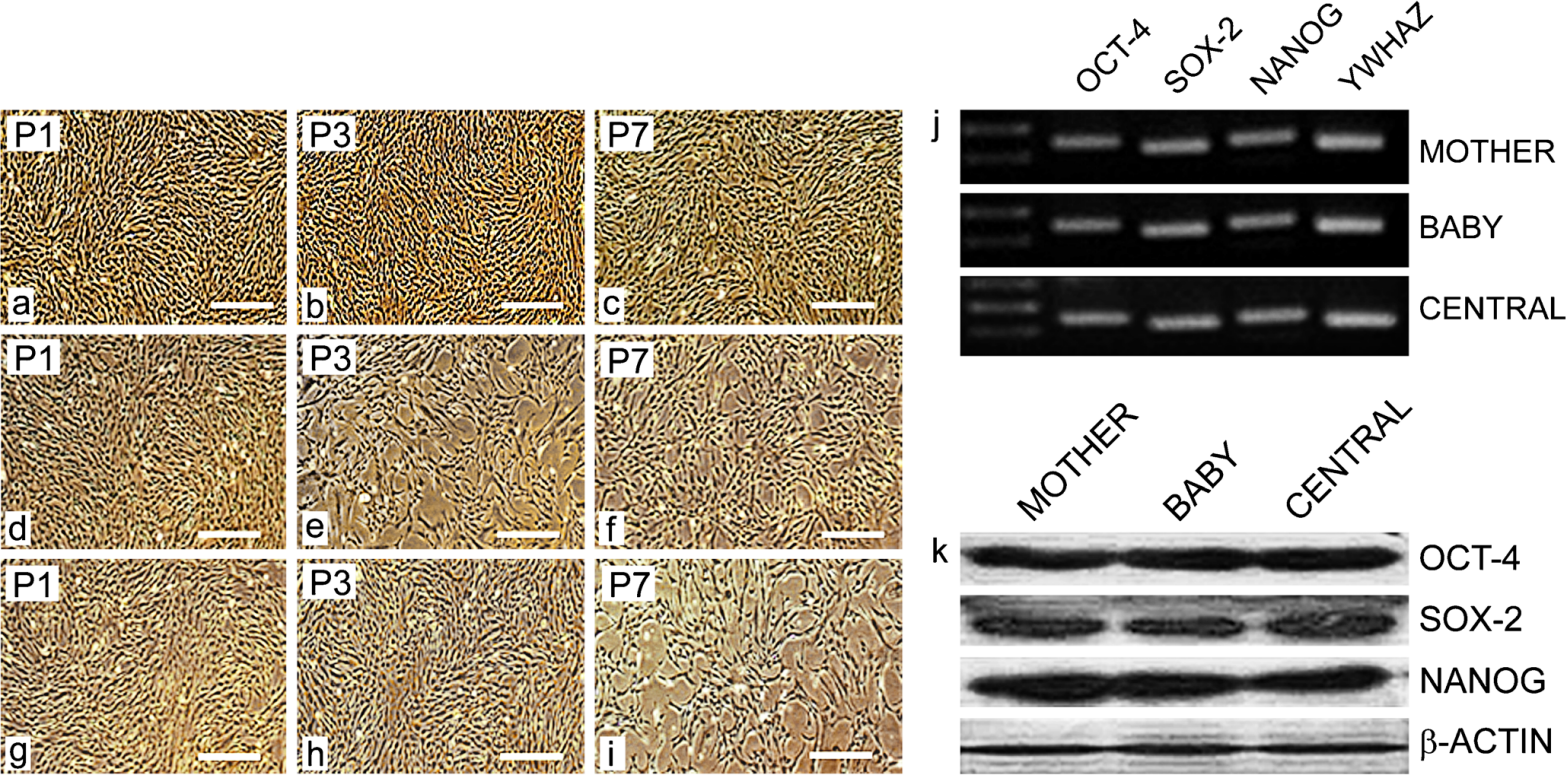
Morphological assessment and pluripotent marker expression. Phase contrast microscopic images of MBC-WJMSCs at passage *P1*, *P3* and *P7* for Mother (**a**–**c**), Baby (**d**–**f**) and Central (**g**–**i**); *Scale bar* 100 μm. **j** Gel electrophoresis images and **k** western blot images of pluripotent markers OCT4, SOX-2 and NANOG at the third passage

Wharton’s jelly 1- to 2-mm explants were firmly adhered to the plastic surface and started showing fibroblastoid-like cell morphology emerging from the sides of the attached tissue. After removal of the explants, primary cells from the three parts of the UC were cultured for 8–10 days until reaching 80% confluence. MBC-WJMSCs were sub-cultured up to the third passage for further comparative analysis. No change in the morphology of the entire three group-derived MSCs was seen even after being sub-cultured for passage 7 (Fig. 2a–i). Further, all the cells positively expressed pluripotency markers OCT-4, SOX-2 and NANOG at mRNA and protein level with no significant differences (Fig. 2j, k).

### Cell surface marker expression (FACS analysis) and population doubling time (PDT)

**Fig. 3:**
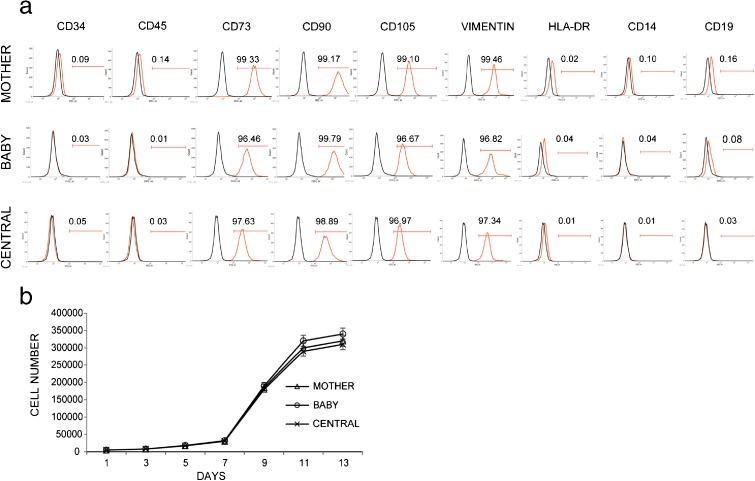
Cell surface marker expression and population doubling time. **a** Cell surface marker expression of MBC-WJMSCs at passage 3. All groups showed positive expression for mesenchymal markers CD90, CD105 and CD73 but were negative for CD34, CD45, CD19, CD14 and HLA-DR. *Gray lines* represent the isotype; *red lines* the corresponding CD marker expression. **b** Population doubling time shown by MBC-WJMSCs at passage 3. A total of 5000 cells per well were seeded in 24-well plates and harvesting was carried out every other day. All groups were experimented on in triplicate

Cells from all three experimental groups were characterized for the expression of cell surface markers using flow cytometer. MBC-WJMSCs were shown to have strong positive expression for CD90, CD105, CD73 and vimentin but negative expression for CD34, CD45, CD14, CD19 and HLA-DR (Fig. 3a). All three groups shared almost similar CD marker expression. MBC-WJMSCs exhibited comparatively same PDT with no statistical differences. Doubling time was found to be 49.56 ± 0.58 h, 49.30 ± 0.58 h and 51.47 ± 0.60 h for mother, baby and central parts-derived WJMSCs, respectively (Fig. 3b).

### In vitro tri-lineage differentiation

**Fig. 4:**
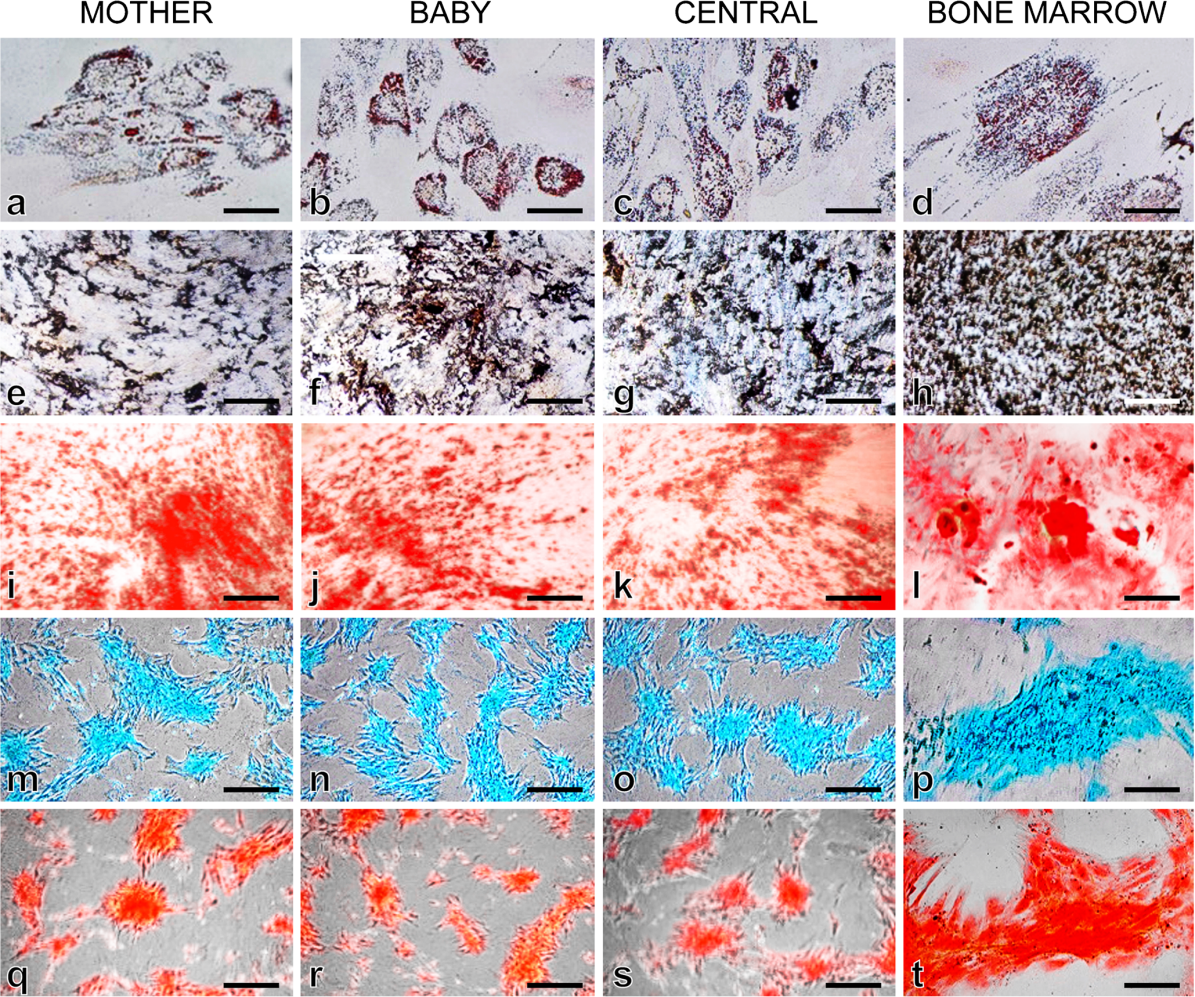
Determination of trilineage differentiation potential of MBC-WJMSCs and BMMSCs. In vitro differentiation potential of MBC-WJMSCs and BMMSCs into adipocytes (Oil Red O, **a**–**d**), osteocytes (Von Kossa, **e**–**h** and Alizarin Red, **i**–**l**), and chondrocytes (Alcian Blue, **m**–**p**, and Safranin-O, q–t); *scale bar* 100 μm

**Fig. 5:**
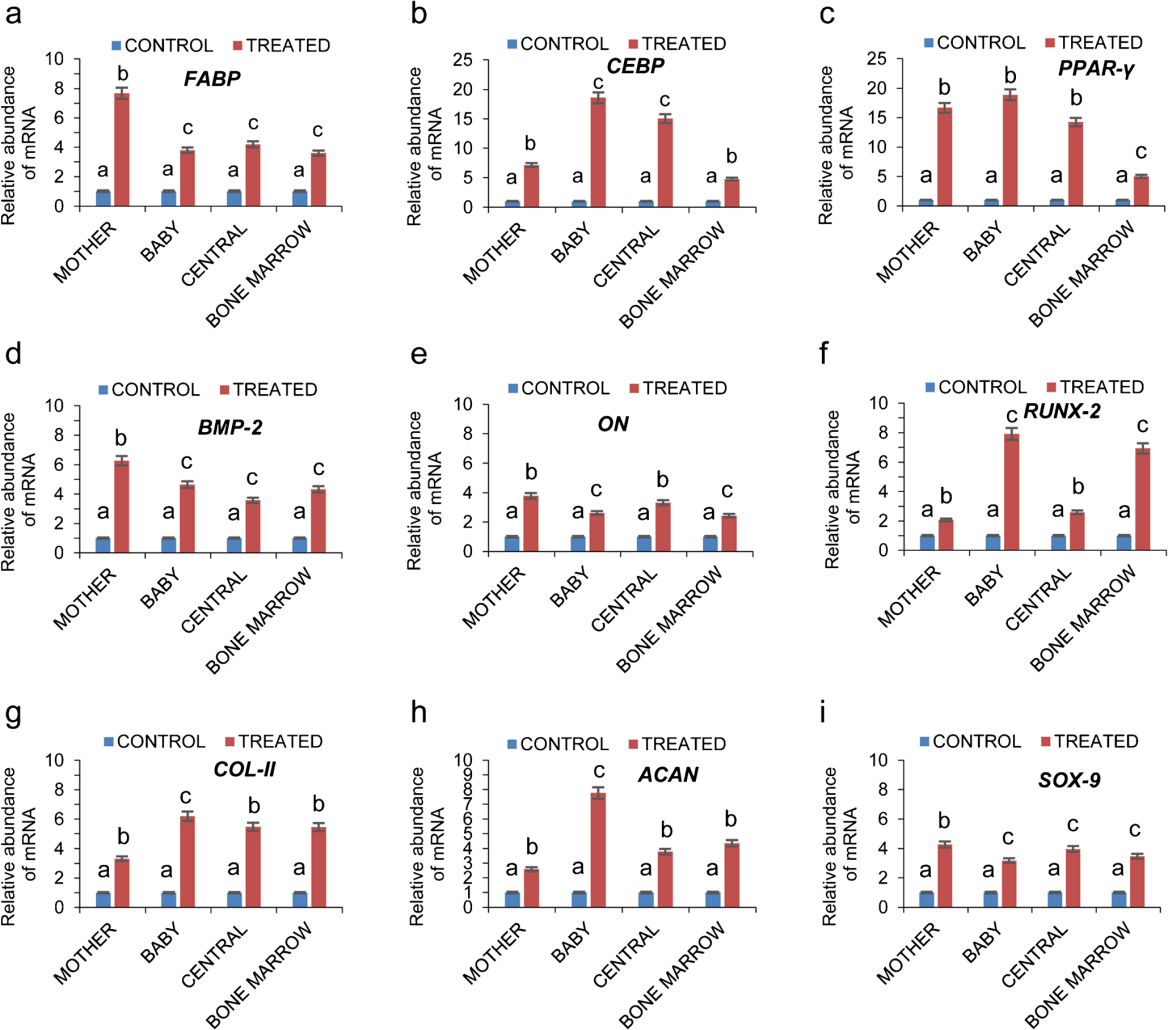
Determination of lineage specific gene expression. **a**–**i** Relative mRNA levels of adipocyte lineage specific genes *FABP*, *PPAR* and *CEBP* (**a**–**c**), osteocyte lineage-specific genes *BMP2*, *ON* and *RUNX-2* (**d**–**f**) and chondrocyte-specific genes *ACAN*, *COL-11* and *SOX-9* (**g**–**i**), respectively. Significant differences were considered when *P* < 0.05. See text for explanation of abbreviations

Under specific in vitro differentiation conditions and induction with lineage-specific differentiation medium for 3 weeks, MSCs from all the experimental groups were successfully differentiated into adipocytes, osteocytes and chondrocytes. Adipose-differentiated MSCs showed synthesis of intracellular lipid droplets confirmed by Oil red O staining (Fig. 4a–d) whereas mineralized nodule formation in osteocyte-differentiated cells were confirmed and positively stained by von Kossa (Fig. 4e–h) and Alizarin red (Fig. 4i–l). Induction with chondrogenesis medium resulted in the deposition of sulfated proteoglycans and glycosaminoglycans that were further stained with Alcian blue (Fig. 4m–p) and Safranin O (Fig. 4q–t). No such staining results were observed in undifferentiated cells from any of the experimental groups (Fig. S1). Furthermore, adipose-differentiated cells expressed lineage-specific markers such as fatty acid binding protein (*FABP*), CCAT/enhancer binding protein alpha (*C/EBP-α*) and peroxisome proliferative activated receptor gamma (*PPAR-γ*) (Fig. 5a–c). Osteocyte-differentiated cells expressed bone morphogenetic protein-2 (*BMP-2*), osteonectin (*ON*) and runt-related transcription factor-2 (*RUNX-2*) (Fig. 5d–f), whereas chondrocyte lineage-differentiated cells expressed collagen-11 (*COL-11*), aggrecan (*ACAN*) and SRY-Box-9 (*SOX-9*) (Fig. 5g–i).

### Hepatogenic differentiation

**Fig. 6:**
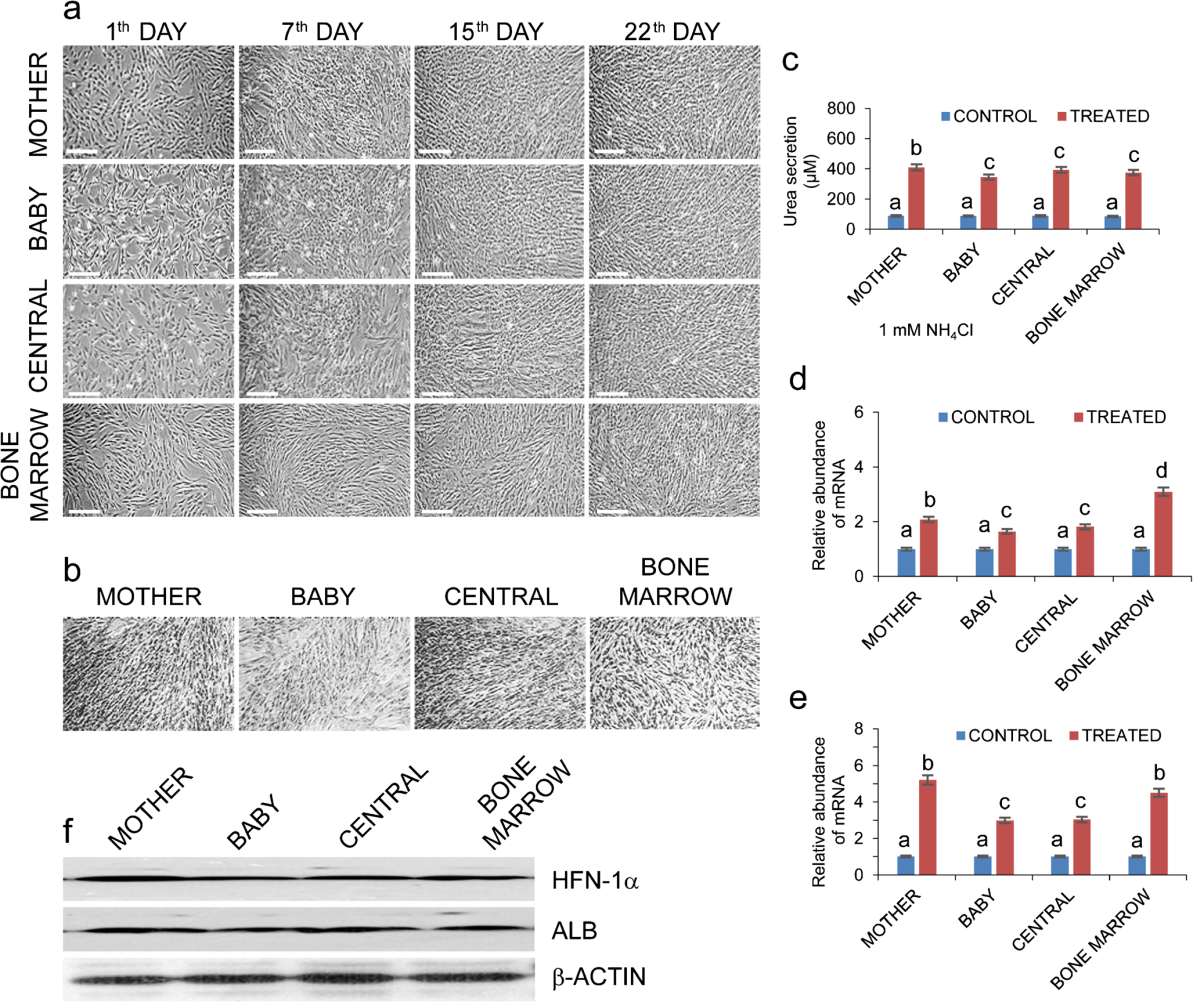
Hepatocyte-like cell differentiation potential of MBC-WJMSCs and BMMSCs. **a** Morphological changes in MBC-WJMSCs and BMMSCs at days 1, 7, 15 and 22 of induction; *scale bar* 100 μm. **b** Differentiated MBC-WJMSCs and BMMSCs showing positive periodic acid-Schiff staining to confirm glycogen storage; *scale bar* 100 μm. **c** Urea secretion by differentiated cells under the influence of 1 mM NH_4_Cl. Values represent the mean ± standard error of the estimate of the mean value of urea secretion in μM obtained in triplicate. **d**, **e** Differentiated MBC-WJMSCs and BMMSCs showing positive expression for *HNF4A1* and *ALB* genes. **f** All experimental groups showed positive expression for HNF-1α and ALB proteins by western blotting

To assess the extent of hepatogenic differentiation potential from MBC-WJMSCs and BMMSCs, passage 3 MSCs at 70% confluence were induced in 2% FBS containing hepatocyte differentiation medium for 22 days. Gradual change in the cell morphology was observed during priming and maturation steps when MSCs were exposed to hepatocyte-specific growth factors. With the progression in hepatocyte induction, MSCs and BMMSCs derived from all three parts showed broadened, flattened and polygonal-shaped epithelial cell-like morphology (Fig. 6a). Mother part-derived WJMSCs (M-WJMSCs) showed slightly higher hepatogenic potential as compared to baby (B-WJMSCs) and central (C-WJMSCs) parts. BMMSCs showed significantly higher *HNF-1α* expression in comparison to all other groups, whereas for the *ALB* expression no significant differences were observed among M-WJMSCs and BMMSCs. Differentiated cells from all the groups showed positive PAS staining of glycogen granules present in their cytoplasm (Fig. 6b), although a mild positive expression for PAS staining was also observed from the undifferentiated cells but at a much lower extent than the differentiated MSCs (Fig. S1). In the next experiment, we examined the ability of induced cells to produce urea, which indicates ammonia metabolism by differentiated hepatocytes. Our results revealed that differentiated MBC-MSCs had the ability to produce urea and showed 4.59-, 3.91- and 4.40-fold urea secretion as compared to their untreated counterparts, respectively. Differentiated BMMSCs showed 4.38-fold urea secretion in comparison to untreated cells (Fig. 6c).

**Fig. 7:**
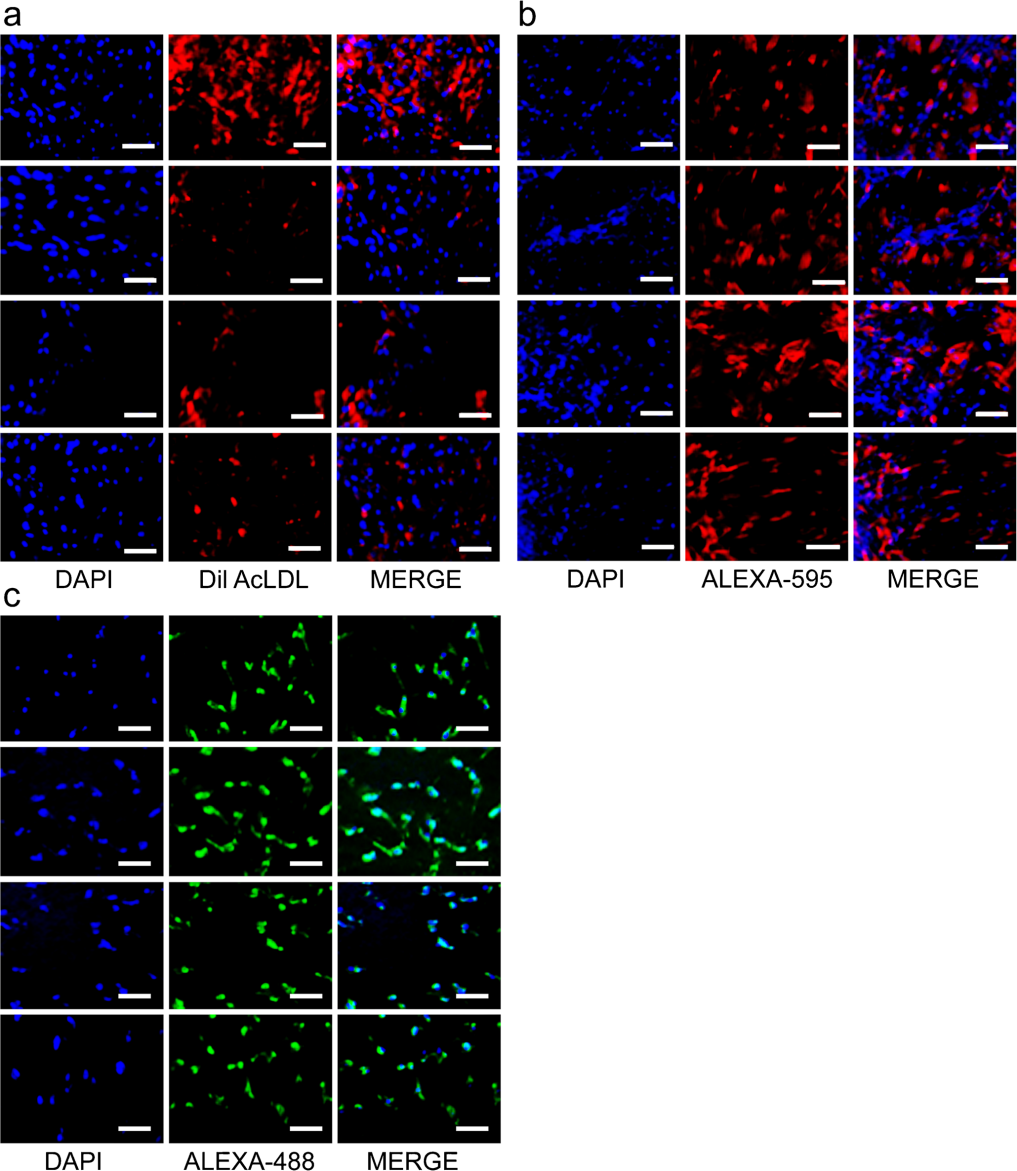
Immunostaining images of differentiated MBC-WJMSCs and BMMSCs. **a** Fluorescent images showing ability of differentiated MBC-WJMSCs and BMMSCs to uptake Dil AcLDL after 4 h incubation at 37 °C; *scale bar* 100 μm. **b**, **c** Immunostaining images showing positive expression for HNF-1α and ALB antibody, respectively, by MBC-WJMSCs after 22 days induction; First, second, third and fourth rows in (**b**, **c**) represent mother, baby, central and bone marrow, respectively; *scale bar* 100 μm

RT-qPCR and western blot analysis revealed positive expression of hepatocyte specific markers hepatocyte nuclear factor-4A1 (HNF-4A1) and ALB from all the experimental groups. BMMSCs showed significantly higher *HNF4A1* expression than the other MSC groups (*P* < 0.05) whereas among WJMSC regions, M-WJMSCs showed highest *HNF4A1* expression (Fig. 6d). Both the M-MSCs and BMMSCs exhibited significantly higher *ALB* (*P* < 0.05) expression than the other two groups (Fig. 6e). B-MSCs exhibited comparatively lesser expression than the other groups at both mRNA and protein level. Functional analysis of all MSC groups revealed positive expression for LDL uptake, PAS staining and urea synthesis assays. Immunocytochemical staining of differentiated cells from all the groups supported RT-qPCR data (Fig. 7b, c) and western blot results showed positive expression of hepatocyte markers (Fig. 6f). Successful differentiation was further confirmed by lipoprotein uptake by differentiated cells, a characteristic feature of hepatocytes (Fig. 7a), whereas untreated cells did not display the same. All differentiated MSCs expressed hepatocyte markers at protein level (Figs. 6f, 7b, c). This confirms that the signal obtained in the induced MSCs was not due to the background signal. To check the specificity of the hepatocyte marker-specific antibodies, untreated cells were also immunostained but displayed no signal (Fig. S2). Collectively, M-MSCs displayed slightly higher hepatocyte differentiation potential when compared to the other groups.

## Discussion

The selection of highly potent MSCs as a therapeutic agent in clinical studies has always been the main motive of stem cell researchers. WJMSCs have been presented as the best source of clinically usable MSCs because of a lack of ethical concerns, easy availability, non-invasive isolation with high yield and greater differentiation and immunomodulatory potential. Moreover, recent research findings have revealed that WJ is the best stem cell source among other compartments of the UC (Subramanian et al. 2015). However, whether WJMSCs isolated from different parts of same-donor UCs constitutes the same mesenchymal characteristics throughout its length is still unclear and no such study has been elucidated until now. Therefore, the present study aimed to compare different parts of UC-derived WJMSCs from the same donor to determine whether or not all parts comprise the same extent of mesenchymal features on the basis of cell surface and pluripotent markers expression, PDT, tri-lineage differentiation potential and transdifferentiation ability into HLCs. The isolation of MSCs using the explant method has been shown to be advantageous over enzymatic methods by limiting possible cellular damage caused by enzymes (Yoon et al. 2013; Ishige et al. 2009). To get rid of the enzymatic effect on the primary cells, the explant attachment method was employed to isolate WJMSCs. MBC-WJMSCs displayed plastic-adherent fibroblast-like morphology when cultured under identical conditions. MSCs did not change their morphology even at longer passages, which shows greater cell stability even under prolonged culturing. This feature of cellular stability even at longer passages holds promising results in obtaining abundant safer cell populations suitable for tissue engineering applications (Chang et al. 2014). Cell surface antigen profiling of MBC-WJMSCs revealed higher expression for the mesenchymal markers CD90, CD105 and CD73, whereas cells were negative with low expression of the hematopoietic markers CD34, CD45, CD14, CD19 and HLA-DR cell surface molecules without any significant differences between the experimental groups. All the experimental groups showed strong positive expression for vimentin. These results are in accordance with previous studies (Shivakumar et al. 2016; Subramanian et al. 2015; Fong et al. 2007; Fong et al. 2010).

For using MSCs in cell-based clinical therapy, cell yield, cell survivability and population doubling time hold central attention as higher cell numbers with greater cell survivability and low population doubling times are taken as the priority. WJMSCS hold all these features to be used as suitable therapeutic agents. Further, the non-enzymatic isolation method resulted in attaining cells with lower PDT when compared to our previous study in which the enzymatic method was used for the isolation of WJMSCs. Similar findings have been reported by Ishige et al. (2009) where the explant attachment method promoted the reduced PDT when compared to the enzymatic digestion method with the same culture conditions. In the present study, the PDT study of MBC-WJMSCs showed a slower growth rate during 1–7 days of culturing while it increased up to 5- to 6-fold when entering the logarithmic growth phase that continued for 9 days. In the stationary phase, all the experimental groups displayed the same stagnant rate. Furthermore, MSCs derived from all the parts shared almost the same PDT without significant differences. Similar growth characteristics from all the parts indicate the presence of valuable WJMSCs throughout the length of the whole UC. Further, the stemness of the isolated cells was confirmed by the expression of pluripotent markers OCT-4, SOX-2 and NANOG at the mRNA and protein level with no significant differences between the groups. In our previous study, we obtained similar expression for early transcription factors (Shivakumar et al. 2016). Many other studies have reported pluripotency marker expression in early passages that was diminished with the expansion of the cultures (Kim et al. 2011; Yoon et al. 2013; Tantrawatpan et al. 2013; Subramanian et al. 2015). While comparing pluripotency expression from different compartments of UCs, WJMSCs were shown to exhibit higher expression of these markers when compared to the other parts (Subramanian et al. 2015). Another study has shown that umbilical vein-derived MSCs could not express SOX-2 but was positive for OCT4 and NANOG (Kermani et al. 2008). In evidence of these results, it is confirmed that, as far as the pluripotent markers expression is concerned, WJ is the best MSC source among other parts of the UC and also exhibit unaltered expression by any isolation method (enzymatic digestion method or explant attachment method) or cryopreservation techniques (conventional or programmed). The expression of these markers represents the undifferentiated state of MSCs that are responsible for maintaining self-renewability. Differentiation also constitutes a pivotal role in confirming the stemness of isolated cells along with CD marker profiling and pluripotent expression. Therefore, the tri-lineage differentiation potential of MBC-WJSMCs was assessed by inducing cells into appropriate lineage-specific culture conditions. All the above-discussed WJMSC-related studies have been reported to express tri-lineage differentiation potential by showing lineage-specific positive expression for the markers and staining of the induced cells. However, it was not confirmed whether or not all parts of the same UC-isolated WJMSCs exhibit the same differentiation abilities. Considering these queries, WJMSCs derived from all the parts were induced to the same cultural conditions and for the same duration. In the last few decades, BMMSCs have been considered as the gold standard and used as a positive control to evaluate the lineage-specific differentiation potential of other MSC sources. Therefore, human BMMSCs were also induced to tri-lineages under the same lineage-specific culture conditions as used for inducing WJMSCs. In the present study, staining images of all the adipocyte-, osteocyte- and chondrocyte-differentiated MBC-WJMSCs and BMMSCs showed the same extent of differentiation. MBC-WJMSCs showed higher expression for adipocyte-specific genes. Master transcription genes *PPAR-γ* and *CEBP-α* were highly expressed by B-WJMSCs as compared to its mother and central counterparts. MBC-WJMSCs and BMMSCs displayed differentiation potential for osteocyte and chondrocyte lineages with mixed gene expression. Interestingly, we found slightly higher expression of adipocyte-, osteocyte- and chondrocyte-specific genes in B-WJMSCs.

Fulfillment of mesenchymal standards set by the International society For Cellular Therapy (Dominici et al. 2006) does not fully implicate the use of selected MSCs as suitable therapeutic agents in clinical studies but some additional parameters are also needed to be confirmed before their final proceedings, which include the ability of cells to transdifferentiate and become functionally active under the influence of exogenous growth factors and lineage-specific growth conditions (Anzalone et al. 2010). Keeping these necessities in mind, we further checked the endodermal transdifferentiation potential of MBC-WJMSCs and BMMSCs into HLCs. Our aim was to uncover the best WJMSC source among different WJ regions and, therefore, MSCs derived from all the parts were induced in the same hepatocyte-specific culture conditions. Morphologically, all the experimental groups showed a gradual acquisition of polygonal or cuboidal shapes with the progress in the induction, as shown by previous studies (Patil et al. 2014; Prasajak and Leeanansaksiri 2013; Shivakumar et al. 2016). Due to cellular stimulation with exogenous cytokines, only the morphological similarity with the target lineage does not confirm the successful differentiation. Therefore, expression of hepatocyte markers was assessed at mRNA and protein level. Different protocols have been devised for in vitro hepatogenesis and there are variations between different researchers regarding the selection of media, exogenous cytokines, induction period and finally targeted markers to assess the extent of differentiation (Kamiya et al. 1999; Baharvand et al. 2006; Kim et al. 2011; Lee et al. 2012). However, most of such condition-induced target markers are for characterizing differentiated cells but cannot constitute reliable evidence on their own, as evidenced by some markers like AFP and transthyretin that are not only expressed in liver but also by extra embryonic cells in the yolk sac (Snykers et al. 2009). Moreover, some early markers like CK-18, CK-19 and HNF-4α are also expressed by undifferentiated cells that mimic hepatocytes (La Rocca et al. 2009; Zemel et al. 2009). Therefore, it is foremost required to perform functional assays to declare successful differentiation. In light of these observations, our MSCs could efficiently display functional activities including glycogen storage, as visualized by PAS staining, ammonia metabolization and urea production, as determined by colorimetric assays and LDL uptake. The present study demonstrates the acquisition of all the above-discussed features, thereby confirming the efficiency of the protocol used in this study. Once differentiation is over, with the outcome of functional hepatocytes expressing all the necessary markers at mRNA and protein level, the next step is to check the stability and effect of induced cells in vivo. Zhao and colleagues demonstrated that WJMSCs are not only able to transdifferentiate into functional hepatocytes in vitro but also exhibit the same characteristics in vivo without altering their low immunogenic features (Zhao et al. 2009). These properties strengthen the use of WJMSCs in the development of functional hepatocytes in vitro as well as in vivo. However, immunostaining with HNF-1*α*- and ALB-specific antibodies, LDL uptake and hepatocyte-specific *HNF4A1* and *ALB* genes in the case of M-WJMSCs showed slightly increased expression as compared to the other WJMSC groups. This may possibly be due to the secretion of some unknown paracrine factors by the placenta (maternal decidua), as the mother part of WJ is in close vicinity to it. Likewise, small but gradual decreases in the hepatogenicity in the other distant parts (central and baby) were observed. These results are in accordance with recent studies that show that MSCs isolated from chorionic plate and chorionic villi constituents of term placenta express high stromal cell factor and HGF when compared to WJMSCs, which strongly indicates that different parts of the tissue may express different hepatogenic potential (Lee et al. 2012). These two factors are important in the proliferation and differentiation of hepatocytes. BMMSCs also exhibited comparable HLC differentiation potential to that of WJMSCs.

In conclusion, our explant culture-derived MBC-WJMSCs not only showed unaltered morphology even after prolonged passaging, pluripotent marker expression, low PDT, CD marker profiling, tri-lineage differentiation potential and transdifferentiation ability into HLCs but also advocate the presence of compelling WJMSCs throughout the UC length irrespective of its location. These findings strengthen the utility of every fragment of this postnatal discard material (WJ) to be cryopreserved for future use as an auto/allologous stem cell source and prove its supremacy as an important therapeutic agent in cellular therapies.

## Electronic supplementary material

ESM 1 (DOCX 13 kb)

Fig. S1 Adipocyte, osteocyte, chondrocyte and hepatocyte-like cell specific staining images of untreated MBS-WJMSCs and BMMSCs. Tri-lineage specific staining (Oil Red O, Alizarin Red, Von Kossa, Alcian Blue Safranin O and PAS) images of untreated MBC-WJMSCs and BMMSCs; *scale bar* 100 μm. (GIF 26 kb)

High resolution image (TIFF 391 kb)

Fig. S2 Immunostaining images of untreated MBC-WJMSCs and BMMSCs. Immunostaining images of untreated MBC-WJMSCs and BMMSCs showing negative expression of HNF-1α and ALB antibody after 22 days culture; *scale bar* 100 μm. (GIF 19 kb)

High resolution image (TIFF 188 kb)

## References

[CR1] Anzalone R, Lo Iacono M, Corrao S, Magno F, Loria T, Cappello F, Zummo G, Farina F, La Rocca G (2010). New emerging potentials for human Wharton’s jelly mesenchymal stem cells: immunological features and hepatocyte-like differentiative capacity. Stem Cells Dev.

[CR2] Baharvand H, Hashemi SM, Kazemi Ashtiani S, Farrokhi A (2006). Differentiation of human embryonic stem cells into hepatocytes in 2D and 3D culture systems in vitro. Int J Dev Biol.

[CR3] Bongso A, Fong CY (2013). The therapeutic potential, challenges and future clinical directions of stem cells from the Wharton’s jelly of the human umbilical cord. Stem Cell Rev.

[CR4] Chang Z, Hou T, Xing J, Wu X, Jin H, Li Z, Deng M, Xie Z, Xu J (2014). Umbilical cord Wharton’s jelly repeated culture system: a new device and method for obtaining abundant mesenchymal stem cells for bone tissue engineering. PLoS ONE.

[CR5] Choi SA, Lee JY, Kwon SE, Wang KC, Phi JH, Choi JW, Jin X, Lim JY, Kim H, Kim SK (2015). Human adipose tissue-derived mesenchymal stem cells target brain tumor-initiating cells. PLoS ONE.

[CR6] Dominici M, Le Blanc K, Mueller I, Slaper-Cortenbach I, Marini F, Krause D, Deans R, Keating A, Dj P, Horwitz E (2006). Minimal criteria for defining multipotent mesenchymal stromal cells. The International Society for Cellular Therapy position statement. Cytotherapy.

[CR7] Fong CY, Richards M, Manasi N, Biswas A, Bongso A (2007). Comparative growth behaviour and characterization of stem cells from human Wharton’s jelly. Reprod BioMed Online.

[CR8] Fong CY, Subramanian A, Biswas A, Gauthaman K, Srikanth P, Hande MP, Bongso A (2010). Derivation efficiency, cell proliferation, freeze-thaw survival, stem-cell properties and differentiation of human Wharton’s jelly stem cells. Reprod BioMed Online.

[CR9] Friedenstein AJ, Chailakhjan RK, Lalykina KS (1970). The development of fibroblast colonies in monolayer cultures of guinea-pig bone marrow and spleen cells. Cell Tissue Kinet.

[CR10] Ghionzoli M, Repele A, Sartiani L, Costanzi G, Parenti A, Spinelli V, David AL, Garriboli M, Totonelli G, Tian J, Andreadis ST, Cerbai E, Mugelli A, Messineo A, Pierro A, Eaton S, De Coppi P (2013). Human amniotic fluid stem cell differentiation along smooth muscle lineage. FASEB J.

[CR11] Hu Y, Liang J, Cui H, Wang X, Rong H, Shao B, Cui H (2013). Wharton’s jelly mesenchymal stem cells differentiate into retinal progenitor cells. Neural Regen Res.

[CR12] Huang P, Lin LM, Wu XY, Tang QL, Feng XY, Lin GY, Lin X, Wang HW, Huang TH, Ma L (2010). Differentiation of human umbilical cord Wharton’s jelly-derived mesenchymal stem cells into germ-like cells in vitro. J Cell Biochem.

[CR13] Ishige I, Nagamura-Inoue T, Honda MJ, Harnprasopwat R, Kido M, Sugimoto M, Nakauchi H, Tojo A (2009). Comparison of mesenchymal stem cells derived from arterial, venous, and Wharton’s jelly explants of human umbilical cord. Int J Hematol.

[CR14] Kamiya A, Kinoshita T, Ito Y, Matsui T, Morikawa Y, Senba E, Nakashima K, Taga T, Yoshida K, Kishimoto T, Miyajima A (1999). Fetal liver development requires a paracrine action of oncostatin M through the gp130 signal transducer. EMBO J.

[CR15] Kermani AJ, Fathi F, Mowla SJ (2008). Characterization and genetic manipulation of human umbilical cord vein mesenchymal stem cells: potential application in cell-based gene therapy. Rejuvenation Res.

[CR16] Kim MJ, Shin KS, Jeon JH, Lee DR, Shim SH, Kim JK, Cha DH, Yoon TK, Kim GJ (2011). Human chorionic-plate-derived mesenchymal stem cells and Wharton’s jelly-derived mesenchymal stem cells: a comparative analysis of their potential as placenta-derived stem cells. Cell Tissue Res.

[CR17] Kim DW, Staples M, Shinozuka K, Pantcheva P, Kang SD, Borlongan CV (2013). Wharton’s jelly-derived mesenchymal stem cells: phenotypic characterization and optimizing their therapeutic potential for clinical applications. Int J Mol Sci.

[CR18] La Rocca G, Anzalone R, Corrao S, Magno F, Loria T, Lo Iacono M, Di Stefano A, Giannuzzi P, Marasa L, Cappello F, Zummo G, Farina F (2009). Isolation and characterization of Oct-4+/HLA-G+ mesenchymal stem cells from human umbilical cord matrix: differentiation potential and detection of new markers. Histochem Cell Biol.

[CR19] Lee HJ, Jung J, Cho KJ, Lee CK, Hwang SG, Kim GJ (2012). Comparison of in vitro hepatogenic differentiation potential between various placenta-derived stem cells and other adult stem cells as an alternative source of functional hepatocytes. Differentiation.

[CR20] Li CD, Zhang WY, Li HL, Jiang XX, Zhang Y, Tang PH, Mao N (2005). Mesenchymal stem cells derived from human placenta suppress allogeneic umbilical cord blood lymphocyte proliferation. Cell Res.

[CR21] Liang J, Wu S, Zhao H, Li SL, Liu ZX, Wu J, Zhou L (2013). Human umbilical cord mesenchymal stem cells derived from Wharton’s jelly differentiate into cholinergic-like neurons in vitro. Neurosci Lett.

[CR22] Majore I, Moretti P, Stahl F, Hass R, Kasper C (2011). Growth and differentiation properties of mesenchymal stromal cell populations derived from whole human umbilical cord. Stem Cell Rev.

[CR23] McElreavey KD, Irvine AI, Ennis KT, McLean WH (1991). Isolation, culture and characterization of fibroblast-like cells derived from the Wharton’s jelly portion of human umbilical cord. Biochem Soc Trans.

[CR24] Park BW, Jang SJ, Byun JH, Kang YH, Choi MJ, Park WU, Lee WJ, Rho GJ (2014) Cryopreservation of human dental follicle tissue for use as a resource of autologous mesenchymal stem cells. J Tissue Eng Regen Med. 10.1002/term.1945 10.1002/term.1945 25052907

[CR25] Patil R, Kumar BM, Lee WJ, Jeon RH, Jang SJ, Lee YM, Park BW, Byun JH, Ahn CS, Kim JW, Rho GJ (2014). Multilineage potential and proteomic profiling of human dental stem cells derived from a single donor. Exp Cell Res.

[CR26] Prasajak P, Leeanansaksiri W (2013). Developing a new two-step protocol to generate functional hepatocytes from Wharton’s jelly-derived mesenchymal stem cells under hypoxic condition. Stem Cells Int.

[CR27] Shivakumar SB, Bharti D, Subbarao RB, Jang SJ, Park JS, Ullah I, Park JK, Byun JH, Park BW, Rho GJ (2016). DMSO- and serum-free cryopreservation of Wharton’s jelly tissue isolated from human umbilical cord. J Cell Biochem.

[CR28] Snykers S, De Kock J, Rogiers V, Vanhaecke T (2009). In vitro differentiation of embryonic and adult stem cells into hepatocytes: state of the art. Stem Cells.

[CR29] Subramanian A, Fong CY, Biswas A, Bongso A (2015). Comparative characterization of cells from the various compartments of the human umbilical cord shows that the Wharton’s jelly compartment provides the best source of clinically utilizable mesenchymal stem cells. PLoS ONE.

[CR30] Taghizadeh RR, Cetrulo KJ, Cetrulo CL (2011). Wharton’s jelly stem cells: future clinical applications. Placenta.

[CR31] Tantrawatpan C, Manochantr S, Kheolamai P, U-Pratya Y, Supokawej A, Issaragrisil S (2013). Pluripotent gene expression in mesenchymal stem cells from human umbilical cord Wharton’s jelly and their differentiation potential to neural-like cells. J Med Assoc Thailand.

[CR32] Ullah I, Subbarao RB, Kim EJ, Bharti D, Jang SJ, Park JS, Shivakumar SB, Lee SL, Kand D, Byun JH, Park BW, Rho GJ (2016). In vitro comparative analysis of human dental stem cells from a single donor and its neuronal differentiation potential evaluated by electrophysiology. Life Sci.

[CR33] Wang HS, Hung SC, Peng ST, Huang CC, Wei HM, Guo YJ, Fu YS, Lai MC, Chen CC (2004). Mesenchymal stem cells in the Wharton’s jelly of the human umbilical cord. Stem Cells.

[CR34] Wang XY, Lan Y, He WY, Zhang L, Yao HY, Hou CM, Tong Y, Liu YL, Yang G, Liu XD, Yang X, Liu B, Mao N (2008). Identification of mesenchymal stem cells in aorta-gonad-mesonephros and yolk sac of human embryos. Blood.

[CR35] Wu LF, Wang NN, Liu YS, Wei X (2009). Differentiation of Wharton’s jelly primitive stromal cells into insulin-producing cells in comparison with bone marrow mesenchymal stem cells. Tissue Eng Part A.

[CR36] Xu M, Zhang B, Liu Y, Zhang J, Sheng H, Shi R, Liao L, Liu N, Hu J, Wang J, Ning H, Liu T, Zhang Y, Chen H (2014). The immunologic and hematopoietic profiles of mesenchymal stem cells derived from different sections of human umbilical cord. Acta Biochimi Biophysica Sin.

[CR37] Yoon JH, Roh EY, Shin S, Jung NH, Song EY, Chang JY, Kim BJ, Jeon HW (2013). Comparison of explant-derived and enzymatic digestion-derived MSCs and the growth factors from Wharton’s jelly. Biomed Res Int.

[CR38] Zemel R, Bachmetov L, Ad-El D, Abraham A, Tur-Kaspa R (2009). Expression of liver-specific markers in naive adipose-derived mesenchymal stem cells. Liver Int.

[CR39] Zhao Q, Ren H, Li X, Chen Z, Zhang X, Gong W, Liu Y, Pang T, Han ZC (2009). Differentiation of human umbilical cord mesenchymal stromal cells into low immunogenic hepatocyte-like cells. Cytotherapy.

