# Supplementary material for: Comparative analysis of human Wharton’s jelly mesenchymal stem cells derived from different parts of the same umbilical cord
Source: Cell Tissue Res. 2017 Dec 4;372(1):51–65. doi: 10.1007/s00441-017-2699-4 (PMC5862947; doi:10.1007/s00441-017-2699-4)
Supplement: Supplementary file 1 — (DOCX 13 kb) [file 441_2017_2699_MOESM1_ESM.docx]

**Table S1.** List of antibodies, dilution and manufacturer details.

| **Antibody** | **Dilution** | **Manufacturer** |
| --- | --- | --- |
| Goat anti-Oct-3/4 | 1:200 | Santa Cruz Biotechnology |
| Rabbit anti-Sox-2 | 1:200 | Santa Cruz Biotechnology |
| Goat anti-Nanog | 1:200 | Santa Cruz Biotechnology |
| Goat anti-hepatocyte nuclear factor 1-alpha | 1:200 | Santa Cruz Biotechnology |
| Goat anti-human serum albumin | 1:200 | Santa Cruz Biotechnology |
| Donkey ant-goat IgG CFL-594 | 1:200 | Santa Cruz Biotechnology |
| Donkey ant-goat IgG CFL-488 | 1:200 | Santa Cruz Biotechnology |
| Rabbit anti-β actin | 1:1000 | Cell Signaling Technology |
| Horseradish peroxidase (HRP)-conjugated donkey anti-goat IgG | 1:10,000 | Santa Cruz Biotechnology |
| Horseradish peroxidase (HRP)-conjugated donkey anti-rabbit IgG | 1:10,000 | Santa Cruz Biotechnology |
