# Supplementary figures and images for: Comparative analysis of human Wharton’s jelly mesenchymal stem cells derived from different parts of the same umbilical cord
Source: Cell Tissue Res. 2017 Dec 4;372(1):51–65. doi: 10.1007/s00441-017-2699-4 (PMC5862947; doi:10.1007/s00441-017-2699-4)

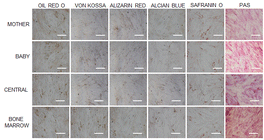

Supplement: Supplementary file 2 — Adipocyte, osteocyte, chondrocyte and hepatocyte-like cell specific staining images of untreated MBS-WJMSCs and BMMSCs. Tri-lineage specific staining (Oil Red O, Alizarin Red, Von Kossa, Alcian Blue Safranin O and PAS) images of untreated MBC-WJMSCs and BMMSCs; scale bar 100 μm. (GIF 26 kb) [file 441_2017_2699_Fig8_ESM.gif]

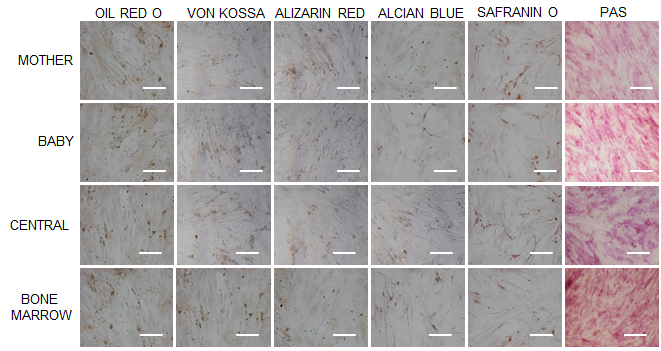

Supplement: Supplementary file 3 — High resolution image (TIFF 391 kb) [file 441_2017_2699_MOESM2_ESM.tif]

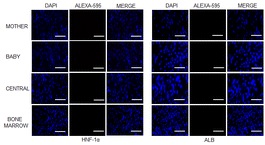

Supplement: Supplementary file 4 — Immunostaining images of untreated MBC-WJMSCs and BMMSCs. Immunostaining images of untreated MBC-WJMSCs and BMMSCs showing negative expression of HNF-1α and ALB antibody after 22 days culture; scale bar 100 μm. (GIF 19 kb) [file 441_2017_2699_Fig9_ESM.gif]

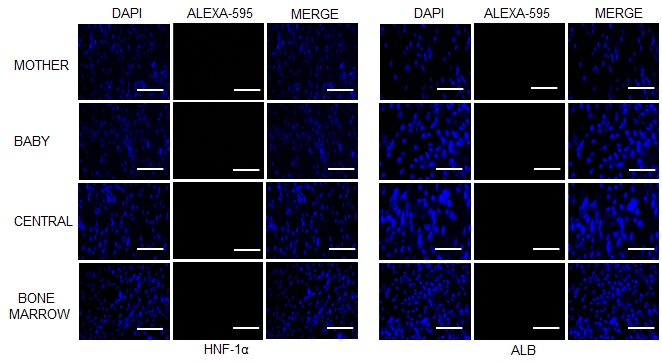

Supplement: Supplementary file 5 — High resolution image (TIFF 188 kb) [file 441_2017_2699_MOESM3_ESM.tif]
